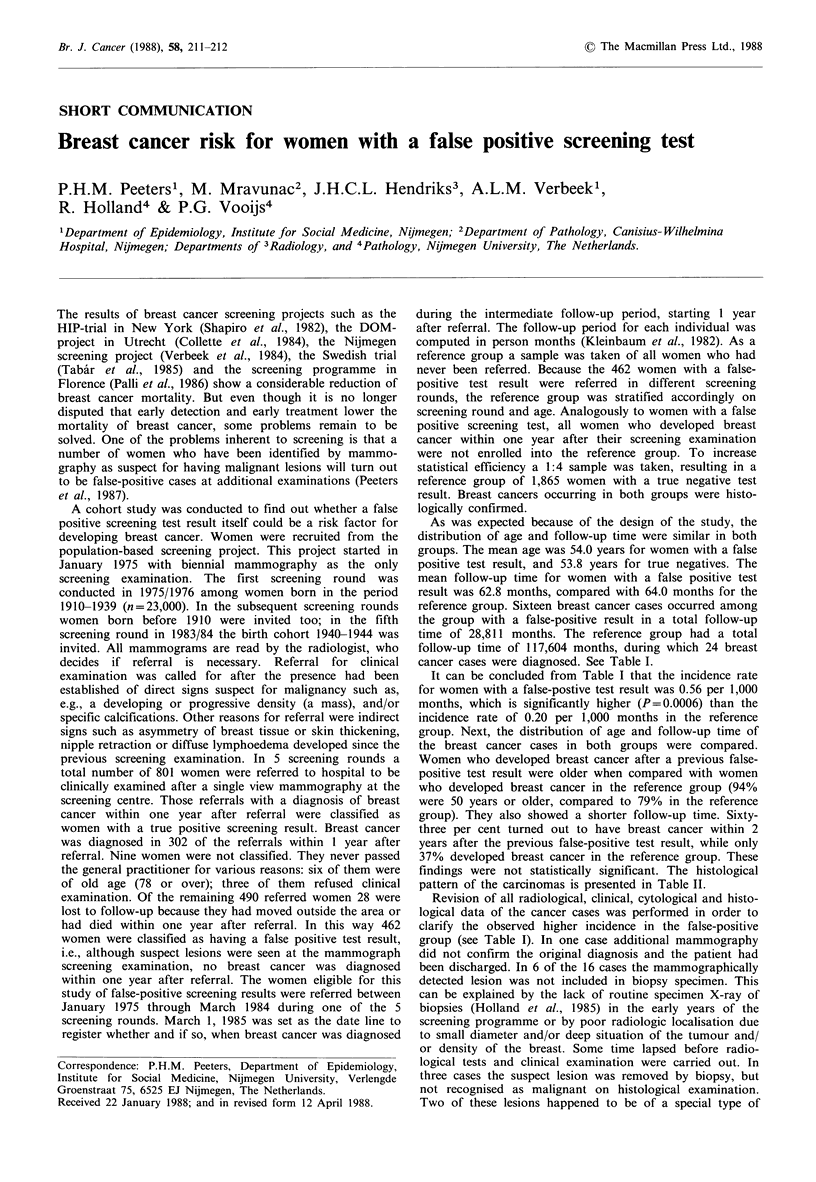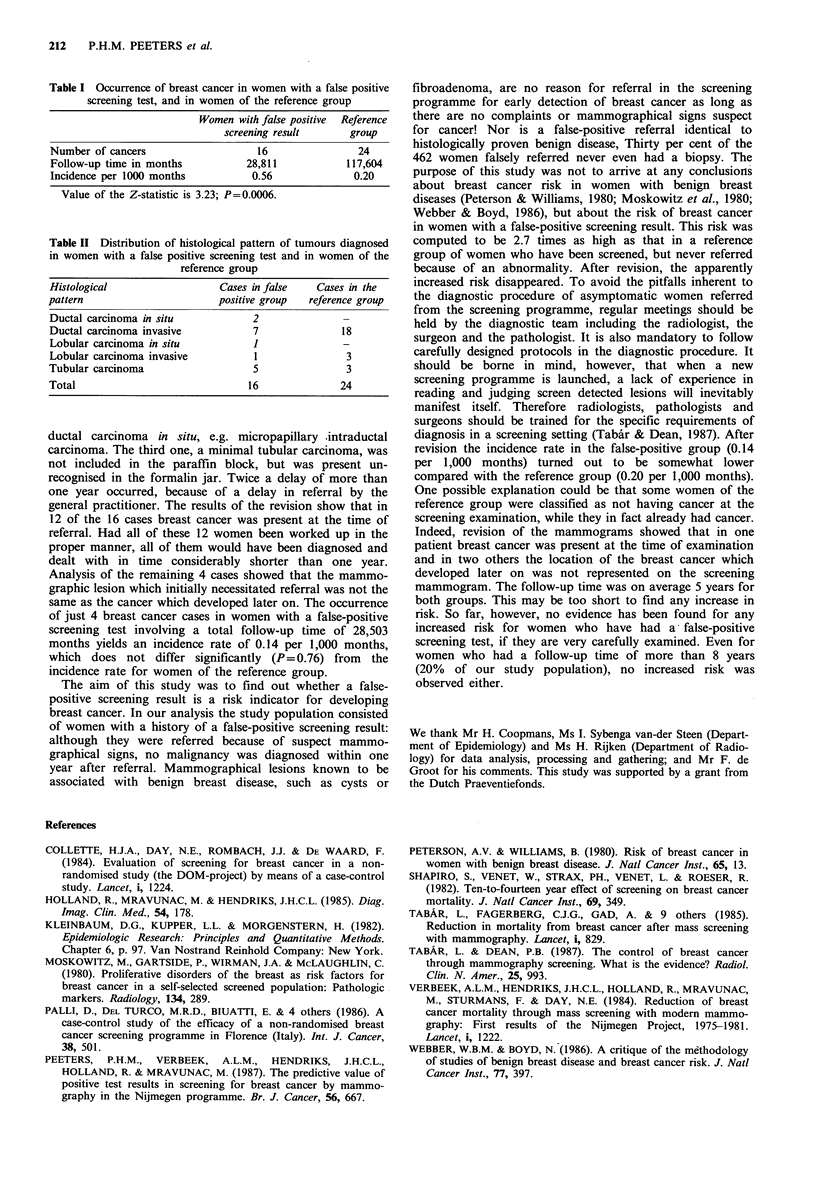# Breast cancer risk for women with a false positive screening test.

**DOI:** 10.1038/bjc.1988.195

**Published:** 1988-08

**Authors:** P. H. Peeters, M. Mravunac, J. H. Hendriks, A. L. Verbeek, R. Holland, P. G. Vooijs

**Affiliations:** Department of Epidemiology, Institute for Social Medicine, Nijmegen, The Netherlands.


Br. J. Cancer (1988), 58, 211–212 © The Macmillan Press Ltd., 1988

SHORT COMMUNICATION

### Breast cancer risk for women with a false positive screening test

P.H.M. Peeters[1], M. Mravunac[2], J.H.C.L. Hendriks[3], A.L.M. Verbeek[1], R. Holland[4] & P.G. Vooijs[4]

[1]*Department of Epidemiology, Institute for Social Medicine, Nijmegen;* [2]*Department of Pathology, Canisius-Wilhelmina Hospital, Nijmegen; Departments of* [3]*Radiology, and* [4]*Pathology, Nijmegen University, The Netherlands.*

The results of breast cancer screening projects such as the HIP-trial in New York (Shapiro *et al*., 1982), the DOM-project in Utrecht (Collette *et al*., 1984), the Nijmegen screening project (Verbeek *et al*., 1984), the Swedish trial (Tabár *et al*., 1985) and the screening programme in Florence (Palli *et al*., 1986) show a considerable reduction of breast cancer mortality. But even though it is no longer disputed that early detection and early treatment lower the mortality of breast cancer, some problems remain to be solved. One of the problems inherent to screening is that a number of women who have been identified by mammography as suspect for having malignant lesions will turn out to be false-positive cases at additional examinations (Peeters *et al*., 1987).

A cohort study was conducted to find out whether a false positive screening test result itself could be a risk factor for developing breast cancer. Women were recruited from the population-based screening project. This project started in January 1975 with biennial mammography as the only screening examination. The first screening round was conducted in 1975/1976 among women born in the period 1910–1939 ($n$=23,000). In the subsequent screening rounds women born before 1910 were invited too; in the fifth screening round in 1983/84 the birth cohort 1940–1944 was invited. All mammograms are read by the radiologist, who decides if referral is necessary. Referral for clinical examination was called for after the presence had been established of direct signs suspect for malignancy such as, e.g., a developing or progressive density (a mass), and/or specific calcifications. Other reasons for referral were indirect signs such as asymmetry of breast tissue or skin thickening, nipple retraction or diffuse lymphoedema developed since the previous screening examination. In 5 screening rounds a total number of 801 women were referred to hospital to be clinically examined after a single view mammography at the screening centre. Those referrals with a diagnosis of breast cancer within one year after referral were classified as women with a true positive screening result. Breast cancer was diagnosed in 302 of the referrals within 1 year after referral. Nine women were not classified. They never passed the general practitioner for various reasons: six of them were of old age (78 or over); three of them refused clinical examination. Of the remaining 490 referred women 28 were lost to follow-up because they had moved outside the area or had died within one year after referral. In this way 462 women were classified as having a false positive test result, i.e., although suspect lesions were seen at the mammograph screening examination, no breast cancer was diagnosed within one year after referral. The women eligible for this study of false-positive screening results were referred between January 1975 through March 1984 during one of the 5 screening rounds. March 1, 1985 was set as the date line to register whether and if so, when breast cancer was diagnosed during the intermediate follow-up period, starting 1 year after referral. The follow-up period for each individual was computed in person months (Kleinbaum *et al*., 1982). As a reference group a sample was taken of all women who had never been referred. Because the 462 women with a false-positive test result were referred in different screening rounds, the reference group was stratified accordingly on screening round and age. Analogously to women with a false positive screening test, all women who developed breast cancer within one year after their screening examination were not enrolled into the reference group. To increase statistical efficiency a 1:4 sample was taken, resulting in a reference group of 1,865 women with a true negative test result. Breast cancers occurring in both groups were histologically confirmed.

As was expected because of the design of the study, the distribution of age and follow-up time were similar in both groups. The mean age was 54.0 years for women with a false positive test result, and 53.8 years for true negatives. The mean follow-up time for women with a false positive test result was 62.8 months, compared with 64.0 months for the reference group. Sixteen breast cancer cases occurred among the group with a false-positive result in a total follow-up time of 28,811 months. The reference group had a total follow-up time of 117,604 months, during which 24 breast cancer cases were diagnosed. See Table I.

It can be concluded from Table I that the incidence rate for women with a false-postive test result was 0.56 per 1,000 months, which is significantly higher ($P$=0.0006) than the incidence rate of 0.20 per 1,000 months in the reference group. Next, the distribution of age and follow-up time of the breast cancer cases in both groups were compared. Women who developed breast cancer after a previous false-positive test result were older when compared with women who developed breast cancer in the reference group (94% were 50 years or older, compared to 79% in the reference group). They also showed a shorter follow-up time. Sixty-three per cent turned out to have breast cancer within 2 years after the previous false-positive test result, while only 37% developed breast cancer in the reference group. These findings were not statistically significant. The histological pattern of the carcinomas is presented in Table II.

Revision of all radiological, clinical, cytological and histological data of the cancer cases was performed in order to clarify the observed higher incidence in the false-positive group (see Table I). In one case additional mammography did not confirm the original diagnosis and the patient had been discharged. In 6 of the 16 cases the mammographically detected lesion was not included in biopsy specimen. This can be explained by the lack of routine specimen X-ray of biopsies (Holland *et al*., 1985) in the early years of the screening programme or by poor radiologic localisation due to small diameter and/or deep situation of the tumour and/or density of the breast. Some time lapsed before radiological tests and clinical examination were carried out. In three cases the suspect lesion was removed by biopsy, but not recognised as malignant on histological examination. Two of these lesions happened to be of a special type of

Correspondence: P.H.M. Peeters, Department of Epidemiology, Institute for Social Medicine, Nijmegen University, Verlengde Groenstraat 75, 6525 EJ Nijmegen, The Netherlands.
Received 22 January 1988; and in revised form 12 April 1988.

**Table I** Occurrence of breast cancer in women with a false positive screening test, and in women of the reference group

| | *Women with false positive screening result* | *Reference group* |
|---|---|---|
| Number of cancers | 16 | 24 |
| Follow-up time in months | 28,811 | 117,604 |
| Incidence per 1000 months | 0.56 | 0.20 |

Value of the $Z$-statistic is 3.23; $P=0.0006$.

**Table II** Distribution of histological pattern of tumours diagnosed in women with a false positive screening test and in women of the reference group

| *Histological pattern* | *Cases in false positive group* | *Cases in the reference group* |
|---|---|---|
| Ductal carcinoma *in situ* | *2* | – |
| Ductal carcinoma invasive | 7 | 18 |
| Lobular carcinoma *in situ* | *1* | – |
| Lobular carcinoma invasive | 1 | 3 |
| Tubular carcinoma | 5 | 3 |
| Total | 16 | 24 |

ductal carcinoma *in situ*, e.g. micropapillary intraductal carcinoma. The third one, a minimal tubular carcinoma, was not included in the paraffin block, but was present unrecognised in the formalin jar. Twice a delay of more than one year occurred, because of a delay in referral by the general practitioner. The results of the revision show that in 12 of the 16 cases breast cancer was present at the time of referral. Had all of these 12 women been worked up in the proper manner, all of them would have been diagnosed and dealt with in time considerably shorter than one year. Analysis of the remaining 4 cases showed that the mammographic lesion which initially necessitated referral was not the same as the cancer which developed later on. The occurrence of just 4 breast cancer cases in women with a false-positive screening test involving a total follow-up time of 28,503 months yields an incidence rate of 0.14 per 1,000 months, which does not differ significantly ($P=0.76$) from the incidence rate for women of the reference group.

The aim of this study was to find out whether a false-positive screening result is a risk indicator for developing breast cancer. In our analysis the study population consisted of women with a history of a false-positive screening result: although they were referred because of suspect mammographical signs, no malignancy was diagnosed within one year after referral. Mammographical lesions known to be associated with benign breast disease, such as cysts or fibroadenoma, are no reason for referral in the screening programme for early detection of breast cancer as long as there are no complaints or mammographical signs suspect for cancer! Nor is a false-positive referral identical to histologically proven benign disease, Thirty per cent of the 462 women falsely referred never even had a biopsy. The purpose of this study was not to arrive at any conclusions about breast cancer risk in women with benign breast diseases (Peterson & Williams, 1980; Moskowitz *et al.*, 1980; Webber & Boyd, 1986), but about the risk of breast cancer in women with a false-positive screening result. This risk was computed to be 2.7 times as high as that in a reference group of women who have been screened, but never referred because of an abnormality. After revision, the apparently increased risk disappeared. To avoid the pitfalls inherent to the diagnostic procedure of asymptomatic women referred from the screening programme, regular meetings should be held by the diagnostic team including the radiologist, the surgeon and the pathologist. It is also mandatory to follow carefully designed protocols in the diagnostic procedure. It should be borne in mind, however, that when a new screening programme is launched, a lack of experience in reading and judging screen detected lesions will inevitably manifest itself. Therefore radiologists, pathologists and surgeons should be trained for the specific requirements of diagnosis in a screening setting (Tabár & Dean, 1987). After revision the incidence rate in the false-positive group (0.14 per 1,000 months) turned out to be somewhat lower compared with the reference group (0.20 per 1,000 months). One possible explanation could be that some women of the reference group were classified as not having cancer at the screening examination, while they in fact already had cancer. Indeed, revision of the mammograms showed that in one patient breast cancer was present at the time of examination and in two others the location of the breast cancer which developed later on was not represented on the screening mammogram. The follow-up time was on average 5 years for both groups. This may be too short to find any increase in risk. So far, however, no evidence has been found for any increased risk for women who have had a false-positive screening test, if they are very carefully examined. Even for women who had a follow-up time of more than 8 years (20% of our study population), no increased risk was observed either.

We thank Mr H. Coopmans, Ms I. Sybenga van-der Steen (Department of Epidemiology) and Ms H. Rijken (Department of Radiology) for data analysis, processing and gathering; and Mr F. de Groot for his comments. This study was supported by a grant from the Dutch Praeventiefonds.

### References

COLLETTE, H.J.A., DAY, N.E., ROMBACH, J.J. & DE WAARD, F. (1984). Evaluation of screening for breast cancer in a non-randomised study (the DOM-project) by means of a case-control study. *Lancet*, **i**, 1224.

HOLLAND, R., MRAVUNAC, M. & HENDRIKS, J.H.C.L. (1985). *Diag. Imag. Clin. Med.*, **54**, 178.

KLEINBAUM, D.G., KUPPER, L.L. & MORGENSTERN, H. (1982). *Epidemiologic Research: Principles and Quantitative Methods.* Chapter 6, p. 97. Van Nostrand Reinhold Company: New York.

MOSKOWITZ, M., GARTSIDE, P., WIRMAN, J.A. & MCLAUGHLIN, C. (1980). Proliferative disorders of the breast as risk factors for breast cancer in a self-selected screened population: Pathologic markers. *Radiology*, **134**, 289.

PALLI, D., DEL TURCO, M.R.D., BIUATTI, E. & 4 others (1986). A case-control study of the efficacy of a non-randomised breast cancer screening programme in Florence (Italy). *Int. J. Cancer*, **38**, 501.

PEETERS, P.H.M., VERBEEK, A.L.M., HENDRIKS, J.H.C.L., HOLLAND, R. & MRAVUNAC, M. (1987). The predictive value of positive test results in screening for breast cancer by mammography in the Nijmegen programme. *Br. J. Cancer*, **56**, 667.

PETERSON, A.V. & WILLIAMS, B. (1980). Risk of breast cancer in women with benign breast disease. *J. Natl Cancer Inst.*, **65**, 13.

SHAPIRO, S., VENET, W., STRAX, PH., VENET, L. & ROESER, R. (1982). Ten-to-fourteen year effect of screening on breast cancer mortality. *J. Natl Cancer Inst.*, **69**, 349.

TABÁR, L., FAGERBERG, C.J.G., GAD, A. & 9 others (1985). Reduction in mortality from breast cancer after mass screening with mammography. *Lancet*, **i**, 829.

TABÁR, L. & DEAN, P.B. (1987). The control of breast cancer through mammography screening. What is the evidence? *Radiol. Clin. N. Amer.*, **25**, 993.

VERBEEK, A.L.M., HENDRIKS, J.H.C.L., HOLLAND, R., MRAVUNAC, M., STURMANS, F. & DAY, N.E. (1984). Reduction of breast cancer mortality through mass screening with modern mammography: First results of the Nijmegen Project, 1975–1981. *Lancet*, **i**, 1222.

WEBBER, W.B.M. & BOYD, N. (1986). A critique of the methodology of studies of benign breast disease and breast cancer risk. *J. Natl Cancer Inst.*, **77**, 397.